# Mapping the spatial distribution of the Japanese encephalitis vector, *Culex tritaeniorhynchus* Giles, 1901 (Diptera: Culicidae) within areas of Japanese encephalitis risk

**DOI:** 10.1186/s13071-017-2086-8

**Published:** 2017-03-16

**Authors:** Joshua Longbottom, Annie J. Browne, David M. Pigott, Marianne E. Sinka, Nick Golding, Simon I. Hay, Catherine L. Moyes, Freya M. Shearer

**Affiliations:** 1 Spatial Ecology & Epidemiology Group, Oxford Big Data Institute, Li Ka Shing Centre for Health Information and Discovery, University of Oxford, Oxford, UK; 2 Institute for Health Metrics and Evaluation, University of Washington, Seattle, WA USA; 3 Oxford Long Term Ecology Laboratory, Department of Zoology, University of Oxford, Oxford, UK; 4 Quantitative & Applied Ecology Group, School of BioSciences, University of Melbourne, Parkville, VIC Australia; 5 Oxford Big Data Institute, Li Ka Shing Centre for Health Information and Discovery, University of Oxford, Oxford, UK

**Keywords:** Species distribution model, Insect vectors, Ecological surveillance, *Culex tritaeniorhynchus*

## Abstract

**Background:**

Japanese encephalitis (JE) is one of the most significant aetiological agents of viral encephalitis in Asia. This medically important arbovirus is primarily spread from vertebrate hosts to humans by the mosquito vector *Culex tritaeniorhynchus*. Knowledge of the contemporary distribution of this vector species is lacking, and efforts to define areas of disease risk greatly depend on a thorough understanding of the variation in this mosquito’s geographical distribution.

**Results:**

We assembled a contemporary database of *Cx. tritaeniorhynchus* presence records within Japanese encephalitis risk areas from formal literature and other relevant resources, resulting in 1,045 geo-referenced, spatially and temporally unique presence records spanning from 1928 to 2014 (71.9% of records obtained between 2001 and 2014). These presence data were combined with a background dataset capturing sample bias in our presence dataset, along with environmental and socio-economic covariates, to inform a boosted regression tree model predicting environmental suitability for *Cx. tritaeniorhynchus* at each 5 × 5 km gridded cell within areas of JE risk. The resulting fine-scale map highlights areas of high environmental suitability for this species across India, Nepal and China that coincide with areas of high JE incidence, emphasising the role of this vector in disease transmission and the utility of the map generated.

**Conclusions:**

Our map contributes towards efforts determining the spatial heterogeneity in *Cx. tritaeniorhynchus* distribution within the limits of JE transmission. Specifically, this map can be used to inform vector control programs and can be used to identify key areas where the prevention of *Cx. tritaeniorhynchus* establishment should be a priority.

**Electronic supplementary material:**

The online version of this article (doi:10.1186/s13071-017-2086-8) contains supplementary material, which is available to authorized users.

## Background

Vector-borne pathogens are spatially confined by the geographical distribution of both their vectors and hosts [1]. Therefore, to identify locations at risk of vector-borne disease transmission, there is a need to identify the vector species responsible for sustaining transmission of the pathogen, and the geographical distribution of these species. Vectors are not uniformly distributed within their overall range and tend to be spatially heterogeneous, resulting in patches of species occurrence [2, 3]. Understanding such spatial variation is essential for discerning locations of high disease risk, with a greater risk of disease transmission being associated with areas of high abundance of pathogen-infected vectors [4, 5].

Japanese encephalitis virus (JEV) is an arbovirus in the family *Flaviviridae* [6] and is one of the most significant aetiological agents of viral encephalitis in Asia [7]. Approximately 3.1 billion people live in Japanese encephalitis (JE) endemic areas within 24 countries, and an estimated 67,000 cases occur annually [8]. Most infections are asymptomatic or cause nonspecific influenza-like illness (~99%); however, within the < 1% of infections resulting in clinical disease, the case fatality rate is approximately 20–30% [8, 9]. Approximately 30–50% of survivors of JE infection experience neuropsychiatric sequelae, which can lead to significant economic loss [9–11], and methods to control the disease, such as pig vaccination, relocation or slaughter, also have a high economic impact [12, 13]. Japanese encephalitis transmission is widespread across temperate areas of Asia, and JEV has recently spread south-east, being reported in Australia [14–17]. The virus exists in an enzootic transmission cycle between mosquitoes and a range of amplifying vertebrate hosts, primarily wading birds of the family *Ardeidae* (herons and egrets) and swine (both wild and domestic) [18–21]. Humans and other mammals such as cattle and horses are dead-end hosts for the virus as they develop an insufficient level of viraemia to re-infect mosquitoes, and the virus is not transmitted directly from person to person [22]. Transmission of the virus to humans from host species requires enzootic vectors (such as *Culex pipiens* [23] which habitually bite birds and other hosts) sustaining the enzootic cycle, as well as bridge vectors which will bite both JEV hosts and humans [24]. An effective vaccine exists, however, even in high vaccine coverage areas, JEV has been shown to circulate in its enzootic stage, presenting a risk to unvaccinated or nonimmune visitors [25, 26].

There is already a comprehensive knowledge base of competent vectors for JE, with the mosquito *Culex tritaeniorhynchus* Giles, 1901 being implicated as the primary vector across much of Asia [23, 27–32]. However, there is little knowledge regarding spatial heterogeneity in the distribution of this species. The distribution of *Culex tritaeniorhynchus* is widespread across South-East Asia and adjacent tropical areas, extends into the Middle East [33–35] and Africa [36–38], and has recently been reported in Europe [39]. The habitat preferences of this species may vary across its wide range, but it is the environments within the limits of JE enzootic transmission that are of importance when considering locations of JE risk. As well as being considered the principal vector of JEV across Asia [40], *Cx. tritaeniorhynchus* is a competent vector of several other arboviruses [41–44]. Temporary and semi-permanent ground pools, and irrigated rice fields with short and sparse vegetation serve as the main larval habitats for *Cx. tritaeniorhynchus* [45, 46], and due to increasing rice production in Asia, there has been an expanding availability of suitable breeding sites for this species [47]. *Culex tritaeniorhynchus* is an opportunistic feeder which is predominantly zoophilic, generally favouring feeding on cattle over pigs [48]. Despite this feeding preference, intensified pig farming has decreased the bridge between humans and vectors, increasing the likelihood of anthropophagy and resulting in high incidence of JE in areas where pig farms are situated close to human dwellings [49]. *Culex tritaeniorhynchus* is exophagic, and although biting periodicity differs across Asia, this species is a night-time feeder showing two peaks in biting time, a few hours after sunset and around midnight [50, 51].

Future efforts to understand the geographical variation in human risk of JEV infection in Asia would benefit from an improved understanding of the spatial distribution of its primary vector. Species distribution models have been used to model other mosquito vectors of medical importance [52–55], and are a useful tool when considering spatial variation in the risk of mosquito-borne disease transmission. Previous distribution models for *Cx. tritaeniorhynchus* have principally focused on specific countries of interest (the Republic of Korea, Masuoka et al. [56] and Saudi-Arabia, Naeem et al. [57]). One study has modelled this species across multiple countries but used a limited dataset of occurrence records due to the amount of published data available at the time (Miller et al. [58]). More data is now available, and this presents an opportunity to improve the modelling approach previously used, and to address issues such as sampling bias. Here we provide a contemporary map showing the environmental suitability for *Cx. tritaeniorhynchus* within JE risk areas, which builds on an existing knowledge of the distribution of this species.

## Methods

### Overview

**Fig. 1:**
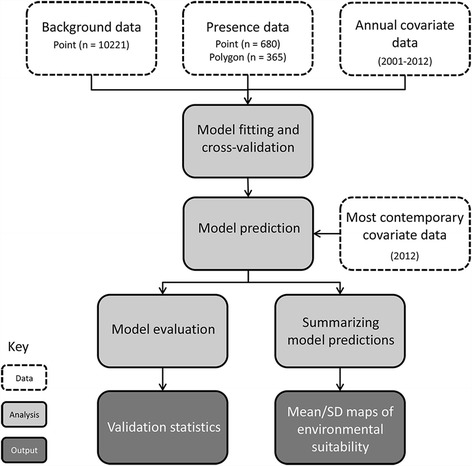
Overview of the methods. *White* boxes describe a data process, *light grey* boxes represent an analysis, and *dark grey* boxes represent final outputs. ‘Point’ data refers to records associated with a location less than 25 km^2^; ‘Polygon’ data refers to records associated with a location greater than 25 km^2^

With the aim of producing a map of environmental suitability for *Culex tritaeniorhynchus* across JE risk areas in Asia, we collated a comprehensive database of geo-positioned occurrence records for this species. Using an ensemble of boosted regression tree (BRT) models, a surface predicting the environmental suitability for *Cx. tritaeniorhynchus* at each 5 × 5 km grid square (pixel) was generated for Southern, Eastern and South-Eastern Asia. Each model utilised a dataset of geo-referenced species occurrence, a background dataset of mosquito survey locations where the species was not reported, and a suite of seventeen gridded annual and synoptic environmental and socio-economic covariates. The final model was used to predict environmental suitability for *Cx. tritaeniorhynchus* at each 5 × 5 km pixel within JE risk areas. A schematic overview of the methods is shown in Fig. 1.

### Occurrence dataset

To expand an existing dataset of *Cx. tritaeniorhynchus* occurrence [58], a literature search was conducted in Web of Science, Scopus and PubMed using the search term “*Culex tritaeniorhynchus”*. The search was limited to articles published between 1^st^ January 2010 and 31^st^ December 2014 and returned 367 unique citations. Initial screening of abstracts filtered out articles which did not report field collections of mosquitoes at a specific location, or the identification of these mosquitoes to the species level. Articles were further limited to those reporting occurrence within Asia, and a more detailed review of the full texts identified 129 articles that met these inclusion criteria.

Mosquito sampling sites were identified within each article and geo-positioned following a variation of existing protocols [59, 60]. Briefly, if coordinates for the sample site were provided within the manuscript, we plotted these coordinates in ArcMap and verified that they matched the sample location description. If a map showing the location of the mosquito collection sites was provided within the article, we digitised this map and extracted coordinates for each of the study sites to obtain the collection location(s). For those articles that did not provide site coordinates or a map, we obtained coordinates for the sample site by searching for the site name in a variety of online gazetteers (Google Maps, GeoNames and OpenStreetMap). All coordinates were recorded in decimal degrees. If the sample location was less than 5 km wide at the widest point, it was treated as a ‘point’. All locations > 5 km were referred to as ‘polygons’, and were split into two classes: (1) ‘administrative polygons’ if a sample site referred to a country’s administrative divisions (administrative levels 1, 2, or 3, as defined by the UN Food and Agriculture Organization’s Global Administrative Units Layer project (GAUL) [61]), and (2) ‘non-administrative polygons’ if a sample site referred to an area > 5 km wide at its widest point which was not a country’s administrative level 1, 2 or 3 division. The appropriate GAUL code was recorded for class 1 polygons, and boundaries were created for class 2 polygons, incorporating the width of the polygon at its widest point. We describe how polygon data were incorporated into our analysis, in detail, in the ‘Model fitting’ section of this paper.

As sampling methodology has been shown to influence the observed presence and abundance of surveyed mosquito species [62, 63], we also recorded the collection method, time of day of collection, and collection month(s) where available, to weight absence records of the species in a location. Data presented for multiple time periods or multiple sites were disaggregated to single time periods and sites where possible. For example, when sampling was performed across a range of years (e.g. 2005–2007), we recorded separate occurrence events for each specific year that *Cx. tritaeniorhynchus* was found (i.e. observations of *Cx. tritaeniorhynchus* at a single location in the years 2005 and 2007 were recorded as two separate occurrences). For studies involving multiple collection sites, an entry was made for each unique site reporting presence; if it was unclear which sampling site was linked to the presence of the species, a polygon was created to encompass all sampling sites to capture the uncertainty in the precise location. Each record was linked to a sampling year to match the observation with the appropriate land cover data available for each year between 2001 and 2012.

*Culex tritaeniorhynchus* presence data were also obtained from the online repositories GBIF (the Global Biodiversity Information Facility http://www.gbif.org/) and VectorMap [64]. Data obtained from online repositories originate mainly from museum records and public reporting of field surveys, and there are many potential errors associated with these sources such as inaccurate geo-positions [65]. We performed spatial validation to ensure the accuracy of this data by overlaying geo-positioned points with a raster distinguishing land from water, and removing any records outside of the land area. We assigned records with uncertainty surrounding the true collection location to the next highest level of geographic precision (as indicated within the ‘CoordinateUncertaintyInMeters’ and ‘coordinateUncertaintyInMeters’ fields within VectorMap and GBIF respectively). Studies which did not separately identify *Cx. tritaeniorhynchus* from the rest of the *Culex vishnui* subgroup were excluded. Records obtained from online repositories were restricted to those reporting occurrences within Asia, and replicate coordinate/year combinations were removed to avoid duplication.

We performed a spatial and temporal standardisation of the final presence dataset to remove any duplicate records, retaining only one occurrence record within each pixel (5 × 5 km) or polygon per the calendar year (as per Kraemer et al. [55] and Moyes et al. [66]). Records without a collection date were assigned a pseudo-collection year by selecting a year at random from the distribution of collection years across our temporally referenced dataset. The final presence dataset contained *Cx. tritaeniorhynchus* occurrence records obtained during the years 1928 to 2014 inclusive. A histogram showing the temporal distribution of our occurrence data is provided as Additional file 1: Figure S1.

### Background dataset

Boosted regression tree (BRT) models utilise binary classification tree algorithms and therefore require both species presence and absence data. When true absence data is not available for a species, background data (also known as pseudo-absence data) can be used. Phillips et al. [67] show that bias in sampling effort can lead to environmental bias in the resulting model and that if such bias is not accounted for, ‘a fitted model might be closer to a model of survey effort than to a model of the true distribution of a species’. The same study showed that the use of carefully selected background data points, which reflect some of the same spatial sampling biases as the presence data, can improve model performance compared to using randomly selected background data. To address both strong spatial biases in survey effort and lack of available absence data, we assembled a background dataset consisting of information on the presence of other mosquito species reported across our study extent. The records within this dataset are subject to similar sampling bias to the occurrence data and are used to expose the model to the range of environments sampled by mosquito surveys using trapping methods similar to those used by studies reporting *Cx. tritaeniorhynchus* (i.e. CDC light traps, resting in/outdoors and man biting trapping techniques).

Other *Culex* species share bionomics with *Cx. tritaeniorhynchus* and surveys for this genus overlap in their sampling design, but unfortunately, data for *Culex* species alone did not provide sufficient coverage across our study extent. We, therefore, used data from surveys of all available mosquito genera as our background dataset, providing better spatial coverage and incorporating potential sources of sample bias within our presence data. Survey data on the presence of *Aedes aegypti* and *Ae. albopictus* obtained from Kraemer et al. [60] were combined with data on the presence of *Anopheles* mosquitoes obtained from the Malaria Atlas Project spatial repository [68]; data on the presence of other *Culex* spp. and all available *Aedes* spp. obtained from VectorMap [64]; data available on the presence of mosquitoes within each of the 41 known mosquito genera obtained from GBIF and data on *Aedes*, *Anopheles* and *Culex* spp. presence from PopBio [69]. All background data were subject to the same spatial and temporal standardisation as the presence data, records were obtained for Asia only, and only point records (locations less than 25 km^2^) and mosquitoes identified to the species level were retained.

### Land-cover and explanatory variables

Carefully selected environmental datasets greatly contribute to the predictive power of species distribution models [70, 71], and are increasingly used to aid predictions of disease vector distributions [52–55, 66]. Seventeen 5 × 5 km gridded surfaces covering a range of environmental (*n* = 15) and socio-economic (*n* = 2) covariates hypothesised to influence the distribution of *Cx. tritaeniorhynchus* were included in our model (summarised in Table 1).

**Table 1:** Covariates used in the model. The table contains information on the source, data type, and temporal resolution of the covariates used in the model

Temporal resolution	Covariate	Source
Synoptic	Land surface temperature (LST) day (Mean) Land surface temperature (LST) day (Standard deviation) Land surface temperature (LST) night (Mean) Land surface temperature (LST) night (Standard deviation)	Gap-filled MODIS LST data [81]
	Tasselled cap wetness (Mean) Tasselled cap wetness (Standard deviation) Tasselled cap brightness (Standard deviation)	Gap-filled MODIS satellite data [76]
	SRTM Elevation	Shuttle Radar Topography Mission [83]
Annual (2001–2012)	Closed shrublands (Proportional cover) Open shrublands (Proportional cover) Woody savannas (Proportional cover) Grasslands (Proportional cover) Permanent wetlands (Proportional cover) Croplands (Proportional cover) Cropland natural vegetation mosaic (Proportional cover) Urban and built up (Proportional cover) Barren or sparsely populated (Proportional cover)	MODIS land cover product [74]

Previous studies have shown that land cover is an important factor in habitat suitability for mosquito species [72, 73]. To account for any changes in land cover throughout our study period, annual surfaces of proportional cell coverage for several land cover classes (Closed Shrublands, Open Shrublands, Woody Savannas, Grasslands, Permanent Wetlands, Croplands, Cropland Natural Vegetation Mosaic, Urban and Built Up, Barren or Sparsely Populated) were derived from the International Geosphere-Biosphere land cover classification available within the MODIS MCD12Q1 dataset [74] and aggregated to 5 × 5 km grid cells.

*Culex tritaeniorhynchus* eggs are unable to withstand desiccation [75], making particularly arid areas environmentally unsuitable. We used mean and standard deviation surfaces for Tasselled Cap Wetness (TCW), and a standard deviation surface for Tasselled Cap Brightness (TCB), derived from NASA’s moderate resolution imaging spectrometer (MODIS) satellite imagery [76] to quantify aridity. These surfaces were generated from the original 1 × 1 km dataset and had been gap-filled to model values for areas missing data due to cloud cover using the algorithm of Weiss et al. [77].

Due to the known influences of temperature on the survival of this species [78–80], separate Land Surface Temperature (LST) (daytime and night-time) synoptic mean and standard deviation surfaces were derived from MODIS 8-daily images spanning the period 2000–2014 [81] which were first gap-filled to remove missing values [77] and then aggregated to generate synoptic surfaces [82]. An elevation surface was generated by aggregating the original 90 m spatial resolution dataset obtained from NASA’s Shuttle Radar Topography Mission (SRTM) [83] to 5 × 5 km cells consistent with our other covariates [82]. Covariates previously showed to be highly correlated (correlation coefficients of |*ρ*| > 0.7) were excluded from our model [84].

### Model fitting

We implemented an ensemble BRT model to predict environmental suitability within JE transmission risk areas [17]. Boosted regression tree models combine both regression trees, and boosting (iteratively combining a group of simple models) algorithms to build a linear combination of many trees [85], and have been used to predict the distributions of a number of diseases and disease vectors [52, 55, 66, 84, 86]. Boosted regression trees excel at identifying complex interactions between explanatory variables, and demonstrate strong predictive power when compared to other modelling approaches [87, 88].

We fitted 200 sub-models, each of which was trained to a separate bootstrap of our occurrence and background data, subject to a constraint that the bootstrap contained a minimum of 30 occurrences and 30 background records. If any record selected for an individual bootstrap was linked to a polygon location, a single 5 × 5 km pixel from within that polygon was selected at random. In this way, different pixels from within each polygon were used for each model run within the ensemble, and the uncertainty in the precise location of the record could be accounted for. That is, if the environments within a polygon were highly variable then the variation in the covariate data provided to the different sub-models was also greater, resulting in higher variation in the model outputs. This technique results in a Monte Carlo simulation integrating uncertainty in the spatial location of the true sample site, assuming that the likelihood of mosquito sampling is equal across all pixels. Each sub-model was fitted using the gbm.step procedure in the dismo R package [89] to identify, by cross-validation, the number of trees that maximised predictive capacity in the held-out dataset. The remaining hyperparameters of the BRT algorithm were: tree complexity = 4, learning rate = 0.005, bag fraction = 0.75, cross-validation folds = 10, step size = 10. As the number of background records was considerably higher than the number of occurrence records, we adjusted the weight of background records in each sub-model so that their weighted sum was equal to the weighted sum of occurrence records. This procedure has been shown to increase the model’s ability to discriminate between presence and background data [90].

Environmental values for the locations of each occurrence and background data point were extracted from the covariate data surfaces. In addition, land cover values were extracted for the year matching each occurrence and background data point between 2001 and 2012 (e.g. a presence record obtained during a 2008 collection was assigned land cover class values from the 2008 MODIS land cover surfaces) (73.43% of occurrence records were obtained *via* sampling between 2001 and 2012). Records obtained from collections performed before 2001 (22.07% of occurrence records) were assigned the 2001 land cover values, and records from collections after 2012 were assigned 2012 land cover values (4.5% of occurrence records). Each sub-model prediction was made using the most contemporary land cover class covariates (i.e. 2012).

### Model prediction and evaluation

We calculated the mean predicted value of environmental suitability of the 200 sub-models for each 5 × 5 km pixel within our study extent. Model performance was analysed using the area under the receiver operator curve (AUC) statistic [91] under ten-fold cross validation. The cross-validation process separates the data set into ten subsets containing approximately the same number of occurrence and background points. The sub-model is then iteratively trained using nine of the data subsets, and the performance in predicting the withheld data is evaluated by statistics for AUC, Kappa [92], sensitivity, specificity and the proportion correctly classified (PCC). An AUC value was calculated for each sub-model, given as the mean of the cross-validated AUC across all ten folds. During cross-validation, we used a pairwise distance sampling procedure to prevent the inflation of the evaluation statistics due to spatial sorting bias in the cross-validation subsets [93]. This pairwise distribution sampling procedure results in a lower AUC but is more reliable as the value is not inflated. The AUCs were then averaged across the 200 sub-models to provide an overall estimate of predictive performance in the ensemble. The remaining statistics were also generated for each sub-model, and a mean calculated: (i) Kappa statistic, showing the degree of agreement between the prediction and the observed truth (presence records), taking account of the fact that some of these classifications may have happened simply by chance; (ii) sensitivity, given as the proportion of species observations correctly predicted; (iii) specificity, given as the proportion of background data points correctly predicted; and (iv) PCC, given as the proportion of sites in which the model correctly predicted whether the species occurred or were unobserved. A PCC of 0 means that the model predicted all background points as occurrences/*vice versa*, and a PCC of 1 means that all occurrence and background points were correctly predicted.

### Relative influence of covariates

The relative influence of each covariate used in this study was quantified based on its ability to explain variance in the training dataset. A relative influence (%) was calculated as the sum of the number of times a particular variable was selected for splitting a regression tree in each sub-model, weighted by the squared improvement to the overall model averaged over all trees in the sub-model [94].

### Masking

The final model prediction was masked by the known limits of JE risk, as defined by the Centers for Disease Control and Prevention (CDC) [17].

## Results

**Fig. 2:**
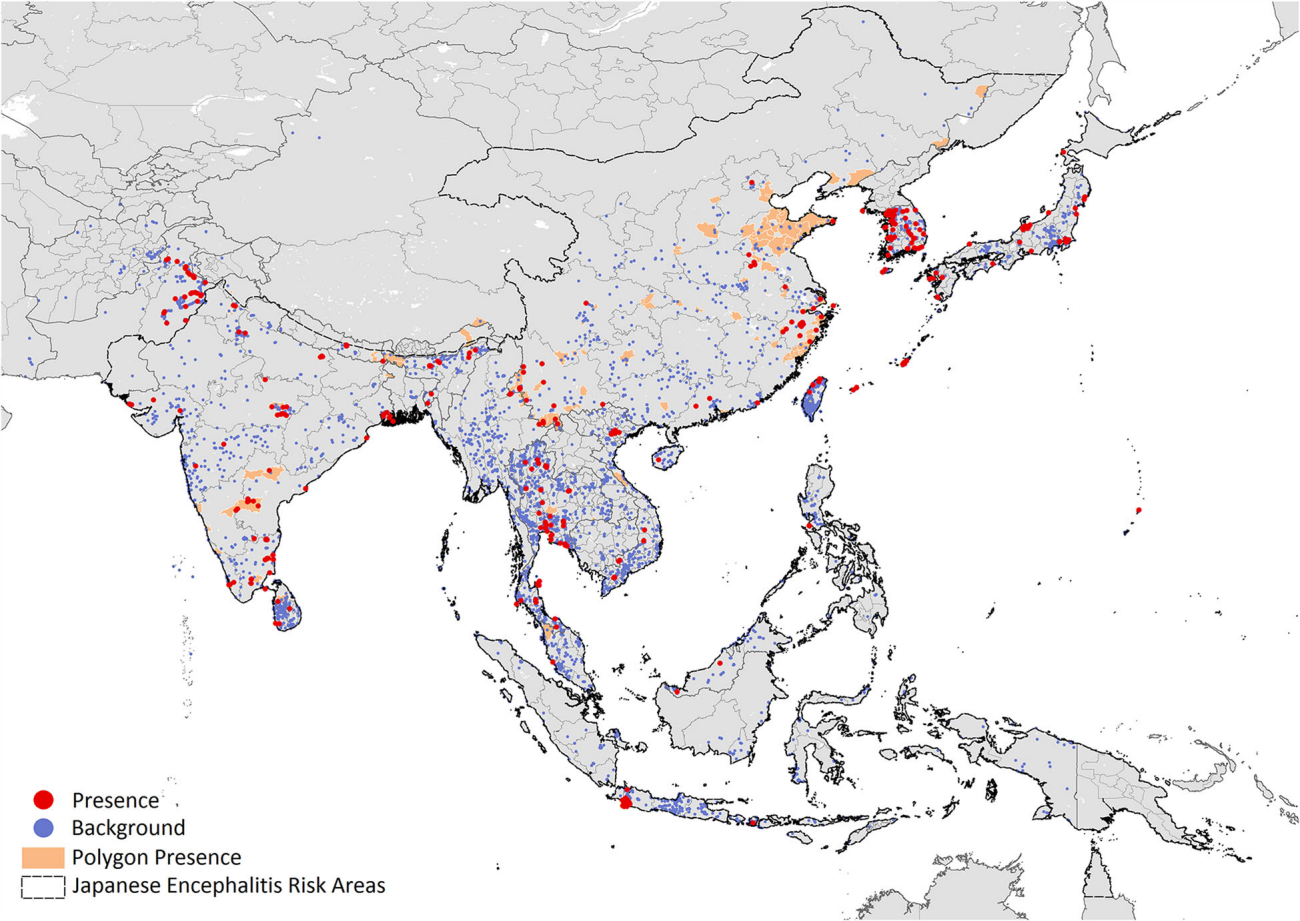
Location of presence and background data used in the model. The map shows the *Culex tritaeniorhynchus* presence points (*red*) and background mosquito species points (*blue*) within the study extent. An extent for Japanese encephalitis limits is also shown (*black*), as published online by the CDC, and provided on an unrestricted basis [17]

A total of 1,045 *Cx. tritaeniorhynchus* spatially and temporally unique presence records were identified, consisting of 680 points and 365 polygons. The background dataset comprised of 10,211 records, representative of 250 mosquito species. The spatial distribution of the presence and background data used to train and fit the model is shown in Fig. 2. All occurrence data obtained from our literature search has been provided as a supplement to this publication (see Additional file 2: Table S1), to ensure reproducibility.

**Fig. 3:**
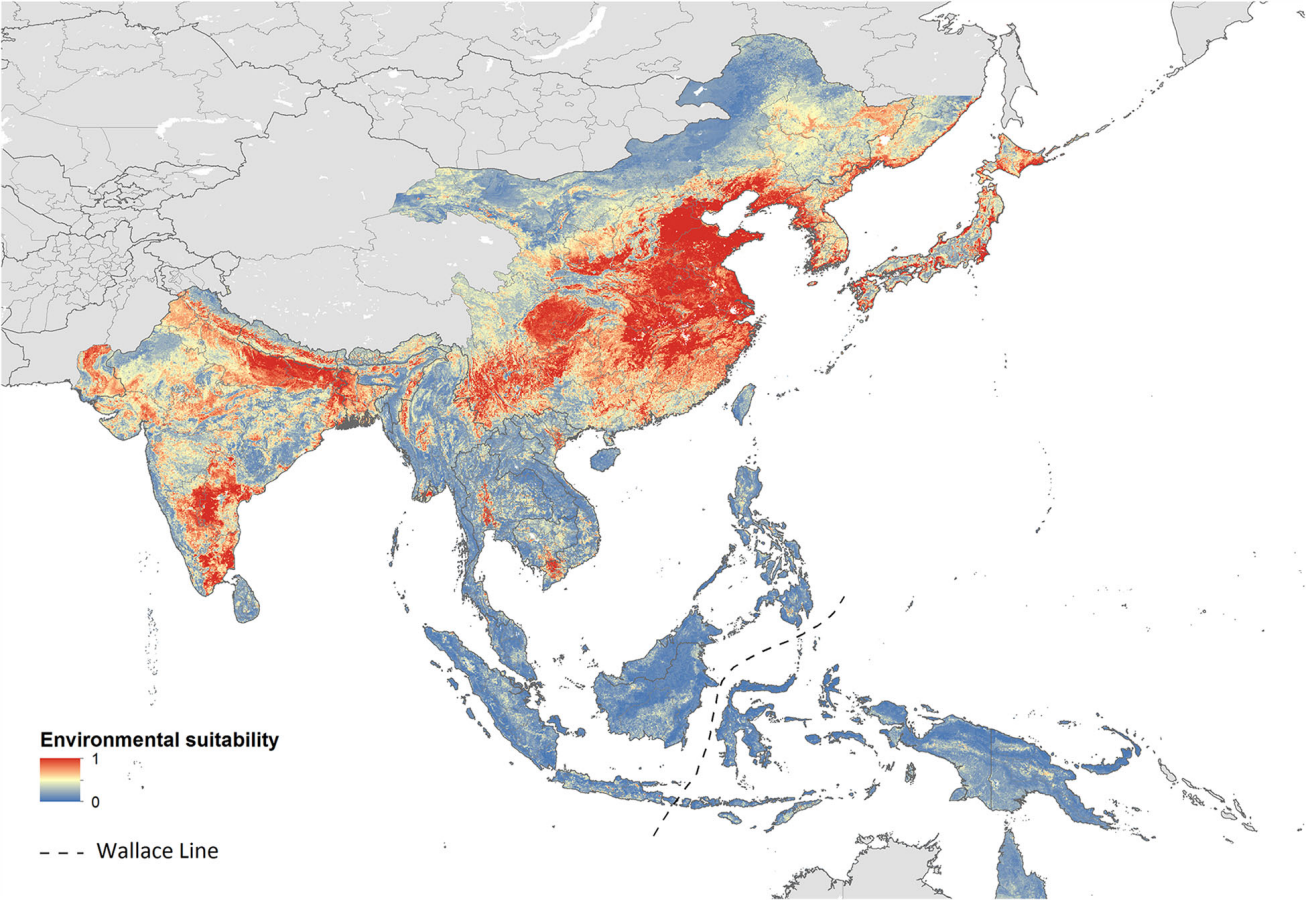
Predicted environmental suitability for *Culex tritaeniorhynchus* within areas at risk of Japanese encephalitis transmission. The map shows the predicted relative environmental suitability for *Culex tritaeniorhynchus* at each 5 × 5 km gridded cell within the limits of Japanese encephalitis [17], on a scale of low environmental suitability (0) to high environmental suitability (1.0)

The model prediction of the environmental suitability for *Cx. tritaeniorhynchus* at each 5 × 5 km pixel within the JE risk area is displayed in Fig. 3, and a GeoTIFF of this output is provided as Additional file 3: Geospatial Data S1 so that readers can explore areas of interest in more detail. Overall, ten-fold cross-validation statistics for the model ensemble resulted in an AUC of 0.71 ± 0.002 standard error, demonstrating moderate predictive power (an AUC of 0.5 is equivalent to ‘random draw’ prediction). Other validation statistics returned for the ensemble were (i) Kappa = 0.40 ± 0.009 standard error, (ii) sensitivity = 0.667 ± 0.006 standard error, (iii) specificity = 0.732 ± 0.006 standard error, and (iv) PCC = 0.70 ± 0.005 standard error. A map of model uncertainty (standard deviation on the logit scale) is provided within Additional file 4: Figure S2.

The model output was restricted to the reported range of JE [17]. The predictions of high *Cx. tritaeniorhynchus* environmental suitability primarily occurs above the Wallace line, and suggest that *Cx. tritaeniorhynchus* is predominantly an Asiatic species, with highly suitable environments located across India, Nepal and China.

In China, the areas with high predicted environmental suitability encompass most of the known presence locations (Fig. 2), and suitability is also predicted in several provinces for which presence data is lacking (Gansu Sheng in northwest China, Shaanxi Sheng in northwest China, Hunan Sheng in south - central China and Henan Sheng in south-central China). Due to the sparse availability of data for Indonesian Borneo (Kalimantan), Sumatra and the Philippines, it remains unclear if areas predicted to be highly suitable are already inhabited or have yet to be colonised by the species.

The predictor with the highest relative influence on the *Cx. tritaeniorhynchus* environmental suitability model was Land Surface Temperature day (standard deviation), which is expressed as the standard deviation in MODIS 8-daily images spanning the period 2000–2014. Land Surface Temperature night (mean) and Land Surface Temperature day (mean) had the third and fourth highest relative influence on the model, with SRTM Elevation having the second highest relative influence. The covariates that proved to be the top predictors, and their relative influence on the model, are given in Additional file 5: Table S2.

## Discussion

We have provided robust estimates for the spatial heterogeneity in *Cx. tritaeniorhynchus* distribution within the limits of JE transmission. Evaluation statistics show that the predictive performance of the model was good, and the resulting predictions of high environmental suitability in India, China and Nepal concur with the high reported incidence of JE in these areas (China, annual incidence of 3.4/100,000; India, 1.5/100,000; Nepal, 2.8/100,000 [8]). Alongside high predictions of environmental suitability in India, China and Nepal, our model has predicted varying levels of suitability for *Cx. tritaeniorhynchus* within the other countries listed by the CDC as at risk of Japanese encephalitis virus infection (Fig. 3) demonstrating a potential source of variation in JE risk at a subnational scale. Our model also helps to highlight regions within countries that have a high environmental suitability for *Cx. tritaeniorhynchus*, but a lack of reported species presence, for example, Rajasthan State (North-West India); Sind Province (South-East Pakistan); Hokkaido Prefecture (Northern Japan), and Primorskiy Kray (South-East Russia).

Our covariates aim to account for land cover changes over time by informing the model of the conditions at each occurrence site at the time of sampling using annual data layers. Furthermore, the covariates used here encompass the majority of land cover classes thought to influence the species’ distribution [95, 96]. One limitation, however, is that the annual land cover surfaces used here were only available for the years 2001–2012, and the data included in our model exceed this date range (1928–2014). Despite this limitation, the inclusion of appropriate land cover surfaces is an advancement on the approach used within the previous multi-country *Cx. tritaeniorhynchus* modelling study, where a static, proportional rice coverage surface was the only land cover class used [58]. We also improved previous predictions of environmental suitability for *Cx. tritaeniorhynchus* by adopting methods to account for spatial uncertainty within the area sampled. The previous multi-country prediction of the spatial distribution of *Cx. tritaeniorhynchus* [58] utilised the centroid coordinates for administrative polygons, ignoring the uncertainty surrounding the true collection location, and their model, therefore, did not account for the high levels of diversity amongst environments in large areas. The inclusion of both point and polygon records here, and iteratively subsampling these polygons, enabled us to include much greater data coverage in China, increasing the volume of species occurrence data available to the model by over seven-fold (1,045 presence records in our study *vs* 148 in the previous work [58]). The covariates used here do not capture all of the potential sources of variation that may influence mosquito habitat suitability (factors such as predation and intraspecific competition are hard to quantify); however, the utilization of more than one land cover class ensured that the influence of vegetation on habitat suitability was more fully accounted for in our model. The spatial resolution of our covariates (5 × 5 km) also does not account for micro-habitats which may result in increased environmental suitability [3].

Another constraint in our modelling process was the lack of *Cx. tritaeniorhynchus* absence data. We, therefore, constructed a presence-only data model using a carefully selected background dataset, capturing biases in our presence data, to improve model performance [67]. Future model predictions of the environmental suitability for *Cx. tritaeniorhynchus* would benefit from further sampling in areas lacking in presence data where JEV has been identified, or from the public release of mosquito survey data already obtained within these areas. The use of a carefully selected background dataset within this study presents another methodological improvement on the previous multi-country *Cx. tritaeniorhynchus* modelling study [58], which, through the use of a Maximum Entropy technique, assigned background data randomly across their study extent thus not accounting for potential sampling bias within their presence dataset.

The lack of *Cx. tritaeniorhynchus* occurrence records in several provinces with highly suitable environments in China (Gansu Sheng in northwest China, Shaanxi Sheng in northwest China, Hunan Sheng in south central China and Henan Sheng in south-central China), and throughout much of Nepal (Fig. 3), suggest that there is either a lack of mosquito sampling or reporting here, or that the species is not occupying the environments identified as suitable within these provinces. If the former is true, our map can be used as a basis for highlighting locations within these provinces where increased surveillance of both mosquito and pathogen should be performed, and if the latter is true, our map can be used to identify areas where prevention of *Cx. tritaeniorhynchus* establishment should be a priority.

Transovarial transmission (vertical transmission from parent to offspring) of JEV in *Cx. tritaeniorhynchus* has been demonstrated within the laboratory [97]. This factor, combined with the ability of the vector to overwinter at the extremes of its range [98, 99] and to disperse large distances [100], present the threat of both *Cx. tritaeniorhynchus* and JEV expanding to and establishing in novel suitable environments. Bird species within the family *Ardeidae* are migratory, adding to the potential for JE to spread to new regions [101, 102]. This study did not aim to model the full global range of *Cx. tritaeniorhynchus,* which extends into the Middle East, Africa and Europe [33–39], but the methods used here could be extended and combined with information on *Ardeidae* migration to predict the potential spread of JEV.

In the last few decades, JEV has expanded its geographic range within Asia [14–16, 103, 104]. Factors influencing this expansion are uncertain, but may include an increase in rice farming (increasing the availability of larval habitats for *Cx. tritaeniorhynchus* [47]), an increase in pig farming (bridging the gap between human-mosquito interaction [49]), potential changes in bird migratory patterns [101, 102], and possible disease spread due to wind-dispersal of infectious mosquitoes [1, 100]. To identify areas suitable for sustaining disease transmission to ensure effective preventative methods are enforced, we must ascertain environments which are suitable for the triad of pathogen, host, and vector. Here, we have identified areas within Asia which are likely to be suitable for mosquito establishment.

The focus of our study was the primary vector *Cx. tritaeniorhynchus*, but other mosquito species have been implicated as primary or secondary vectors of JEV (including other members of the *Culex vishnui* subgroup (C*ulex vishnui* and *Culex pseudovishnui*) [105], *Culex fuscocephala* Theobald [27, 106, 107], *Culex gelidus* Theobald [27, 106, 107] and *Culex whitmorei* Giles [107]). Our map should not, therefore, be interpreted as a map of JE risk. Areas of low environmental suitability for *Cx. tritaeniorhynchus*, which are known areas of JE transmission (such as Malaysia and Indonesia, annual incidence of 1.7–3.7/100,000 [8]), should be investigated to determine the primary and secondary species responsible for transmission in these areas. Understanding JE vector species composition in locations of high JE transmission is vital as mosquito bionomics differ by species, and interventions targeting *Cx. tritaeniorhynchus* may not necessarily apply to vectors exhibiting different behavioural traits [108].

## Conclusion

Our map defines geographic variation in suitability for *Cx. tritaeniorhynchus* within the limits of JE transmission, and thus contributes towards efforts to understand the spatial epidemiology of JE. It can be used to aid predictions of current and future changes in disease distribution. Specifically, this map can be used to inform vector control programs, highlighting areas which would most benefit from the use of insecticides and areas which would be ideal locations for sentinel sites to monitor vector abundance and disease presence. This map, coupled with fine spatial resolution maps of JE distribution if available, can also be used in education campaigns to inform individuals of control methods to prevent vector establishment and disease spread in areas of high environmental suitability for *Cx. tritaeniorhynchus*.

## Additional files

Additional file 1: Figure S1. Temporal distribution of *Culex tritaeniorhynchus* occurrence data. Histogram showing the number of spatially unique *Cx. tritaeniorhynchus* occurrence records per year in our dataset (1928–2014). 73.43% of occurrence records were obtained during the years for which we have annual land cover class layers (2001–2012), as indicated by orange *x*-axis breaks. (.docx) (DOCX 85 kb)
Additional file 2: Table S1. Occurrence data used to fit and train the model. The file contains geo-positioned occurrence data obtained from literature searches and GBIF. An ‘OBJECTID’ is provided for VectorMap data to allow readers to obtain the same records used within this study directly from the source. (.csv) (CSV 709 kb)
Additional file 3: Geospatial Data S1. Model output data for *Culex tritaeniorhynchus*. Mean model output, showing relative environmental suitability for *Culex tritaeniorhynchus*. Pixel values range from 0 (low environmental suitability) to 1 (high environmental suitability). This file can be opened in GIS software such as QGIS (http://www.qgis.org/) or ArcMap (http://www.esri.com/software/arcgis). (.tif) (TIF 9082 kb)
Additional file 4: Figure S2. Map of model uncertainty. Standard deviation values for each pixel were calculated across the model ensemble on the logit scale. Areas from lower to higher standard deviation values are shown. (.docx) (DOCX 1008 kb)
Additional file 5: Table S2. The relative influence of each covariate on the model. The relative influence of each covariate in rank order, including mean (%), 2.5% quantile and 97.5% quantile values. (.docx) (DOCX 14 kb)

## Abbreviations

AUC: Area under the receiver operator curve
BRT: Boosted regression tree
CDC: Centers for Disease Control and Prevention
GBIF: Global Biodiversity Information Facility
JE: Japanese encephalitis
JEV: Japanese encephalitis virus
LST: Land surface temperature
MODIS: Moderate resolution imaging spectrometer
PCC: Proportion correctly classified
SRTM: Shuttle Radar Topography Mission
TCB: Tasselled cap brightness
TCW: Tasselled cap wetness
