# Supplementary material for: Mapping the spatial distribution of the Japanese encephalitis vector, Culex tritaeniorhynchus Giles, 1901 (Diptera: Culicidae) within areas of Japanese encephalitis risk
Source: Parasit Vectors. 2017 Mar 16;10:148. doi: 10.1186/s13071-017-2086-8 (PMC5356256; doi:10.1186/s13071-017-2086-8)

**Additional file 1. Temporal distribution of *Culex tritaeniorhynchus* occurrence data.**

Histogram showing the number of spatially unique *Cx. tritaeniorhynchus* occurrence records per year in our dataset (1928-2014). 73.43% of occurrence records were obtained during the years for which we have annual land cover class layers (2001-2012), as indicated by orange x-axis breaks.


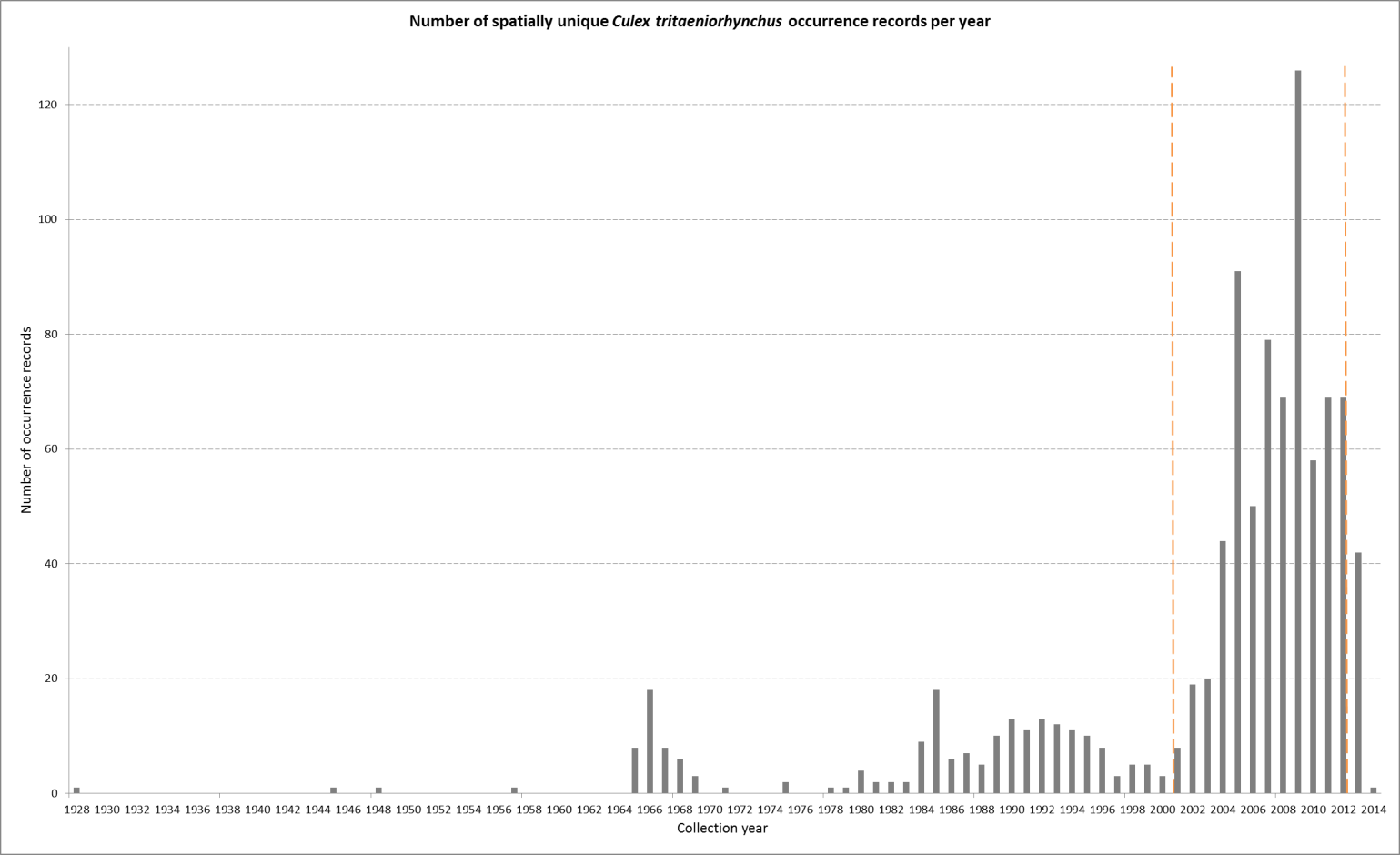

Supplement: Additional file 1: Figure S1. — Temporal distribution of Culex tritaeniorhynchus occurrence data. Histogram showing the number of spatially unique Cx. tritaeniorhynchus occurrence records per year in our dataset (1928–2014). 73.43% of occurrence records were obtained during the years for which we have annual land cover class layers (2001–2012), as indicated by orange x-axis breaks. (.docx) (DOCX 85 kb) [file 13071_2017_2086_MOESM1_ESM.docx]
