# Supplementary material for: Mapping the spatial distribution of the Japanese encephalitis vector, Culex tritaeniorhynchus Giles, 1901 (Diptera: Culicidae) within areas of Japanese encephalitis risk
Source: Parasit Vectors. 2017 Mar 16;10:148. doi: 10.1186/s13071-017-2086-8 (PMC5356256; doi:10.1186/s13071-017-2086-8)

**Additional file 4. Map of model uncertainty.**

Standard deviation values for each pixel were calculated across the model ensemble on the logit scale. Areas from lower to higher standard deviation values are shown.


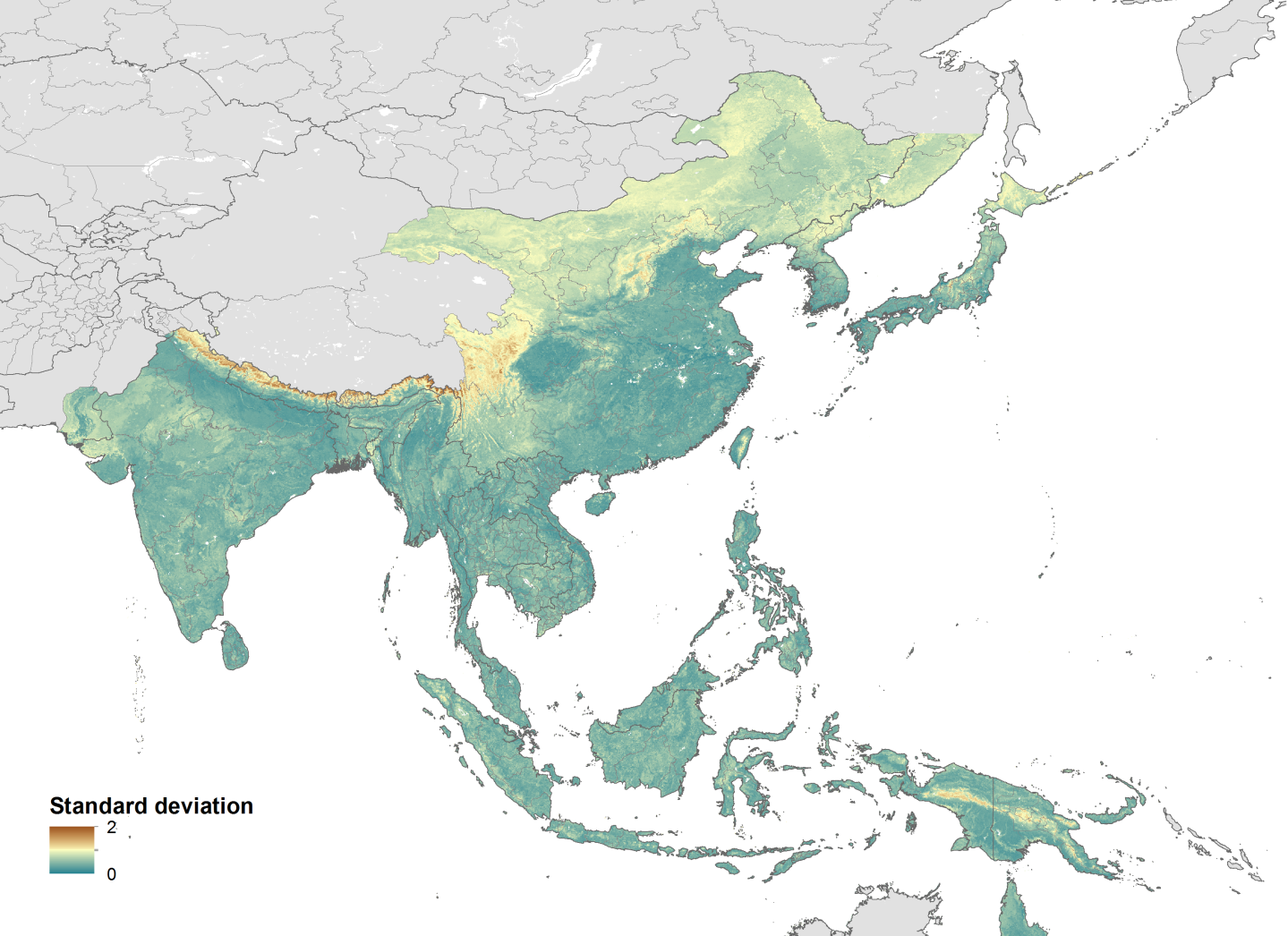

Supplement: Additional file 4: Figure S2. — Map of model uncertainty. Standard deviation values for each pixel were calculated across the model ensemble on the logit scale. Areas from lower to higher standard deviation values are shown. (.docx) (DOCX 1008 kb) [file 13071_2017_2086_MOESM4_ESM.docx]
