# Supplementary material for: Mapping the spatial distribution of the Japanese encephalitis vector, Culex tritaeniorhynchus Giles, 1901 (Diptera: Culicidae) within areas of Japanese encephalitis risk
Source: Parasit Vectors. 2017 Mar 16;10:148. doi: 10.1186/s13071-017-2086-8 (PMC5356256; doi:10.1186/s13071-017-2086-8)
Supplement: Additional file 5: Table S2. — The relative influence of each covariate on the model. The relative influence of each covariate in rank order, including mean (%), 2.5% quantile and 97.5% quantile values. (.docx) (DOCX 14 kb) [file 13071_2017_2086_MOESM5_ESM.docx]

| **Rank** | **Covariate** | **Relative influence** | | |
| --- | --- | --- | --- | --- |
|  |  | **Mean (%)** | **2.5% quantile** | **97.5% quantile** |
| 1 | Land Surface Temperature (LST) day (SD) | 18.377 | 14.265 | 24.144 |
| 2 | SRTM Elevation | 13.213 | 10.180 | 16.880 |
| 3 | Land Surface Temperature (LST) night (Mean) | 10.863 | 9.111 | 13.201 |
| 4 | Land Surface Temperature (LST) day (Mean) | 9.546 | 8.457 | 11.128 |
| 5 | Croplands (PC) | 8.693 | 7.536 | 9.861 |
| 6 | Tasselled Cap Wetness (SD) | 7.837 | 6.781 | 8.943 |
| 7 | Land Surface Temperature (LST) night (SD) | 6.883 | 5.445 | 8.071 |
| 8 | Tasselled Cap Brightness (SD) | 5.478 | 4.588 | 6.566 |
| 9 | Tasselled Cap Wetness (Mean) | 4.741 | 3.903 | 5.738 |
| 10 | Woody Savannas (PC) | 4.004 | 3.094 | 4.923 |
| 11 | Cropland Natural Vegetation Mosaic (PC) | 3.162 | 2.391 | 4.094 |
| 12 | Urban and Built Up (PC) | 2.402 | 1.809 | 3.178 |
| 13 | Permanent Wetlands (PC) | 1.579 | 1.013 | 2.210 |
| 14 | Closed Shrublands (PC) | 1.204 | 0.834 | 1.714 |
| 15 | Grasslands (PC) | 0.940 | 0.625 | 1.280 |
| 16 | Open Shrublands (PC) | 0.730 | 0.440 | 1.017 |
| 17 | Barren or Sparsely Populated (PC) | 0.348 | 0.111 | 0.597 |

**Additional file 5. The relative influence of each covariate on the model.** The most influential covariate is ranked first.

**Abbreviations**

SD = Standard deviation

PC = Proportional cover
